# Poor-Law Nurses and Trade Unions

**Published:** 1920-08-21

**Authors:** John Simonds

**Affiliations:** Secretary. National Poor-Law Officers' Association Incorporated, Norfolk House, Norfolk Street, Strand


### THE EDITOR'S LETTER-BOX.

**Correspondence on all subjects is invited, but we cannot in any way be responsible for the opinions expressed by our correspondents, who must give their name and address as a guarantee of good faith, but not necessarily for publication. Correspondents are reminded that brevity of style and conciseness of statement greatly facilitate early insertion.**

### Poor-Law Nurses and Trade Unions.

*To the Editor of* The Hospital.

Sir,—Poor-Law nurses in different parts of the country are now being urged to join a Trade Union. Without any doubt Trade Unions have proved of great benefit to the employees in most industries, but it is submitted that many Trade Union methods, particularly the strike, ought not to be employed on behalf of nurses engaged in the care of the sick and aged poor. Apart from this objection, it is extremely doubtful whether such methods could be employed effectively on behalf of Poor-Law nurses.

Certainly these nurses do require an organisation to look after their interests, and for many reasons the National Poor-Law Officers' Association Incorporated is the best for their purposes. For a subscription of 5s. a year this Association offers to Poor-Law nurses exactly the same advantages (except that very doubtful one, the strike weapon) as those offered by Trade Unions, the subscriptions to which vary from 17s. 4d. to 26s. a year. A very large number of Poor-Law nurses in England and Wales are already members of the Association or its Approved Society. There are separate sections for Poor-Law nurses in the branches of the Association, and those sections manage their own affairs through committees composed of matrons and nurses elected by the members of the sections. The Executive of the Association has also a Nursing Committee, on which medical officers, matrons, and nurses are represented, dealing entirely with questions affecting the Poor-Law Nursing Service. The National Poor-Law Officers' Association is also closely in touch with the Association of Poor-Law Unions (the Guardians' Association). The Conciliation Council for the Poor-Law Service, established by those two Associations, consists of an equal number of representatives from each of the two organisations.

It will therefore be seen that in the National Poor-Law Officers' Association Poor-Law nurses have all the advantages of a separate organisation of their own, with the additional advantage of having behind it the great weight and experience of the Association's Central Executive and Parliamentary Committee. The Association can justly claim to have done more than any other organisation to improve the pay and general conditions of service of Poor-Law nurses. At its instance the Local Government Board (now Ministry of Health) sanctioned for Poor-Law officers war bonuses in accordance with the scales in operation for Civil Servants, and the majority of nurses in the Poor Law who are now receiving these bonuses have obtained them through the efforts of this Association. During the past eighteen months the Association has successfully represented Poor-Law nurses in no less than sixteen arbitrations relating to claims for war bonus. The reductions in the hours of duty of nurses recently adopted by many Boards of Guardians are mainly due, in the first instance, to the Return of Hours of Duty and Leave of Resident Poor-Law Officials which the Local Government Board called for in 1914 at the instigation of the National Poor-Law Officers' Association, and, latterly, to the Association's propaganda work and the recommendations of the Service Conciliation Council. I should also mention that the National Poor-Law Officers' Association has been in sympathy with the chief aims and objects of the College of Nursing since its inception, and a considerable proportion of the members of the Association's Nurses' Sections are also members of the College.—Yours faithfully,

John Simonds, Secretary.

National Poor-Law Officers' Association Incorporated,
Norfolk House, Norfolk Street, Strand,
August 10, 1920.

